# Identification of novel inhibitors of *Mycobacterium smegmatis* growth through genome-wide overexpression of Cluster P3 mycobacteriophage Xavia genes

**DOI:** 10.1093/g3journal/jkag262

**Published:** 2026-09-16

**Authors:** Anushka Tennakoon, Iresha K Edirisingha, Cole R Jirsa, Ethan Rutledge, Ankita Bhattacharyya, Ella King, Madelyn Moresi, Amara Lovings, Parker R Yarborough, Anna Hoben, Garrett Manns, Toney Ray Gibson, Syed Mohammed Ashfaque Uddin, Yeseul Bae, Natalie Taylor, Taylor L Johnson, Madelyn Futral, Paige Wilson, Jalyn Brown, Zackari L Arbogast, Abigail Brooks, Carson Ward, Courtney Foxworth, Kacey Nguyen, Daphne Fairchild, Deisy Lemus, Hayley White, Holly Craft, Justin Adonis, Kamiya Givan, Sergio Valdivia, Kayci Beth Wallace, Madeline Bent, Shikelya D Chambliss, Dmitri Mavrodi, Danielle M Heller, Ramesh Rijal

**Affiliations:** School of Biological, Environmental, and Earth Sciences, University of Southern Mississippi, Hattiesburg, MS 39406, United States; School of Biological, Environmental, and Earth Sciences, University of Southern Mississippi, Hattiesburg, MS 39406, United States; Department of Science Education, Howard Hughes Medical Institute, Chevy Chase, MD 20815, United States; School of Biological, Environmental, and Earth Sciences, University of Southern Mississippi, Hattiesburg, MS 39406, United States; School of Biological, Environmental, and Earth Sciences, University of Southern Mississippi, Hattiesburg, MS 39406, United States; School of Biological, Environmental, and Earth Sciences, University of Southern Mississippi, Hattiesburg, MS 39406, United States; School of Biological, Environmental, and Earth Sciences, University of Southern Mississippi, Hattiesburg, MS 39406, United States; School of Biological, Environmental, and Earth Sciences, University of Southern Mississippi, Hattiesburg, MS 39406, United States; School of Biological, Environmental, and Earth Sciences, University of Southern Mississippi, Hattiesburg, MS 39406, United States; School of Biological, Environmental, and Earth Sciences, University of Southern Mississippi, Hattiesburg, MS 39406, United States; School of Biological, Environmental, and Earth Sciences, University of Southern Mississippi, Hattiesburg, MS 39406, United States; School of Biological, Environmental, and Earth Sciences, University of Southern Mississippi, Hattiesburg, MS 39406, United States; School of Biological, Environmental, and Earth Sciences, University of Southern Mississippi, Hattiesburg, MS 39406, United States; School of Biological, Environmental, and Earth Sciences, University of Southern Mississippi, Hattiesburg, MS 39406, United States; School of Biological, Environmental, and Earth Sciences, University of Southern Mississippi, Hattiesburg, MS 39406, United States; School of Biological, Environmental, and Earth Sciences, University of Southern Mississippi, Hattiesburg, MS 39406, United States; School of Biological, Environmental, and Earth Sciences, University of Southern Mississippi, Hattiesburg, MS 39406, United States; School of Biological, Environmental, and Earth Sciences, University of Southern Mississippi, Hattiesburg, MS 39406, United States; School of Biological, Environmental, and Earth Sciences, University of Southern Mississippi, Hattiesburg, MS 39406, United States; School of Biological, Environmental, and Earth Sciences, University of Southern Mississippi, Hattiesburg, MS 39406, United States; School of Biological, Environmental, and Earth Sciences, University of Southern Mississippi, Hattiesburg, MS 39406, United States; School of Biological, Environmental, and Earth Sciences, University of Southern Mississippi, Hattiesburg, MS 39406, United States; School of Biological, Environmental, and Earth Sciences, University of Southern Mississippi, Hattiesburg, MS 39406, United States; School of Biological, Environmental, and Earth Sciences, University of Southern Mississippi, Hattiesburg, MS 39406, United States; School of Biological, Environmental, and Earth Sciences, University of Southern Mississippi, Hattiesburg, MS 39406, United States; School of Biological, Environmental, and Earth Sciences, University of Southern Mississippi, Hattiesburg, MS 39406, United States; School of Biological, Environmental, and Earth Sciences, University of Southern Mississippi, Hattiesburg, MS 39406, United States; School of Biological, Environmental, and Earth Sciences, University of Southern Mississippi, Hattiesburg, MS 39406, United States; School of Biological, Environmental, and Earth Sciences, University of Southern Mississippi, Hattiesburg, MS 39406, United States; School of Biological, Environmental, and Earth Sciences, University of Southern Mississippi, Hattiesburg, MS 39406, United States; School of Biological, Environmental, and Earth Sciences, University of Southern Mississippi, Hattiesburg, MS 39406, United States; School of Biological, Environmental, and Earth Sciences, University of Southern Mississippi, Hattiesburg, MS 39406, United States; School of Biological, Environmental, and Earth Sciences, University of Southern Mississippi, Hattiesburg, MS 39406, United States; School of Biological, Environmental, and Earth Sciences, University of Southern Mississippi, Hattiesburg, MS 39406, United States; Wheat Health, Genetics and Quality Research Unit, U.S. Department of Agriculture, Agricultural Research Service, Pullman, WA 99164, United States; Department of Science Education, Howard Hughes Medical Institute, Chevy Chase, MD 20815, United States; School of Biological, Environmental, and Earth Sciences, University of Southern Mississippi, Hattiesburg, MS 39406, United States

**Keywords:** mycobacteriophage, *mycobacterium smegmatis*, cytotoxicity, genome-wide screen

## Abstract

Bacteriophages encode numerous genes with no known function, many of which can affect essential cellular processes when expressed in the bacterial host. For mycobacteriophages, genome-wide overexpression in *Mycobacterium smegmatis* can be used to identify proteins that impair growth. To evaluate the cytotoxic potential of the Cluster P3 phage Xavia, we constructed a plasmid library containing 71 predicted Xavia genes under the anhydrotetracycline-inducible promoter *pTet* and screened this library in a plate-based cytotoxicity assay to measure impacts on *M. smegmatis* growth. Two genes prevented transformant recovery, consistent with toxicity under basal promoter leakiness, and inducible expression of 18 additional genes impaired growth. These inhibitory proteins include structural components; factors involved in DNA metabolism, lysogeny, and lysis; and several proteins with no known function. These results extend functional screening into a lineage of actinobacteriophages that has not previously been characterized, and identify new proteins that warrant further mechanistic analysis.

## Introduction

Bacteriophages have co-evolved with bacterial hosts for billions of years, producing highly mosaic genomes shaped by extensive horizontal gene transfer (Pedulla et al. 2003; Russell and Hatfull 2017). Phage-encoded genes have generated key molecular tools, including restriction enzymes, CRISPR-Cas systems, and regulators of bacterial immunity (Roberts 2005; Makarova et al. 2011; Cong et al. 2013; Mali et al. 2013; Loenen et al. 2014; Ahmed et al. 2018; Hampton et al. 2020; Al-Ouqaili et al. 2025). Many phage proteins act directly on host pathways, which control envelope integrity, DNA replication, and stress responses (Frye et al. 2005; Payne et al. 2009; Darwin 2013; Stone et al. 2019; Dunne et al. 2021; Kim et al. 2024). At the same time, most phage genomes encode large numbers of proteins with no recognizable sequence features (Hatfull 2015, 2020). These “no known function” (NKF) genes represent a reservoir of unexplored activities that may alter host physiology or antimicrobial susceptibility.

To organize the diversity of actinobacteriophage proteins, predicted proteins are grouped into phamilies (phams) based on amino-acid similarity (Cresawn et al. 2011; Gauthier et al. 2022). Previous SEA-GENES overexpression screens showed that cytotoxic NKF genes are often enriched in early lytic regions, which supports roles in host takeover and establishes genome-wide screening as an effective approach for mapping phage gene function (Ko and Hatfull 2018; Heller et al. 2022; Amaya et al. 2023; Heller et al. 2024; Pollenz et al. 2024; Tafoya et al. 2025).

Mycobacteria include important human pathogens such as *Mycobacterium tuberculosis* and nontuberculous mycobacteria, many of which show intrinsic antibiotic tolerance and limited treatment options (Wallis et al. 1999; Nguyen 2016; Boldrin et al. 2020; Goossens et al. 2020). Because direct genetic screening in *M. tuberculosis* is not practical, *M. smegmatis* serves as a well-established surrogate host for identifying conserved vulnerabilities in mycobacterial physiology (Altaf et al. 2010; Lelovic et al. 2020; Sundarsingh T et al. 2020; Sparks et al. 2023). Phage genes that impair *M. smegmatis* growth can identify conserved processes that are broadly essential across mycobacteria (Sparks et al. 2023).

Here, we present a genome-wide overexpression analysis of the Cluster P, Subcluster P3 mycobacteriophage Xavia, a temperate siphovirus encoding 71 genes, of which only 39 have predicted functions. Cluster P phages are genetically distinct from previously examined Cluster K and Cluster F phages and have not previously undergone functional cytotoxicity screening. We identified phage genes that impair *Mycobacterium smegmatis* growth, placed them in functional and phamily context, and compared their distribution to previously screened mycobacteriophages (Heller et al. 2022; Amaya et al. 2023; Pollenz et al. 2024; Tafoya et al. 2025). In total, two Xavia genes prevented consistent recovery of *M. smegmatis* transformants, while overexpression of 18 additional genes produced measurable inducer-dependent cytotoxicity. Altogether these 20 growth inhibitors represent 28% of the Xavia genome. This study extends SEA-GENES functional coverage into a previously untested phage cluster and identifies novel phage gene products with cytotoxic activity.

## Materials and methods

### Growth of mycobacteria and mycobacteriophage

*Mycobacterium smegmatis* mc^2^155 was grown at 37 °C in Middlebrook 7H9 (Difco) broth supplemented with 10% AD (2% wt/vol dextrose [Fisher Chemical], 145 mM NaCl [Fisher Chemical], 5% wt/vol albumin fraction V [Millipore], 0.05% Tween 80 [Fisher Scientific], 10 µg/mL cycloheximide [Acros Organics], and 50 µg/mL carbenicillin [Research Products International]) or on Middlebrook 7H11 (Difco) agar supplemented with 10% AD, 0.5% glycerol (Fisher Scientific), 10 µg/mL cycloheximide, and 50 µg/mL carbenicillin. The competent *M. smegmatis* cells were prepared according to the SEA-GENES Instructor Guide (https://seagenesinstructorguide). A single isolated colony of *M. smegmatis* mc^2^155 was grown overnight (∼ 16 h) in 5 mL of 7H9 complete medium at 220 rpm and 37 °C until the cultures reached an optical density (OD) between 1 and 3. Then, 400 mL of 7H9 complete with Tween 80 (0.05%) was inoculated with the *M. smegmatis* mc^2^155 overnight culture, at a starting OD of 0.005. The new culture was allowed to incubate at 37 °C while shaking at 220 rpm for 16 to 24 h until the culture reached an OD of ∼0.6. Then, the cells were incubated on ice for 1.5 h. The chilled cultures were then centrifuged at 2,000 × *g* for 20 min at 4 °C. The supernatant was discarded, and the cells were washed with 200 mL of ice-cold 10% glycerol 3 times at 2,000 × *g* for 20 min at 4 °C. After the final wash, the cells were resuspended in 1:250 of the original volume with ice-cold 10% glycerol and used in transformation.

To transform cells with pExTra plasmids (Heller et al. 2022), 100 ng of pExTra-Xavia gene plasmid DNA (described below) was mixed with 50 µL of electrocompetent *M. smegmatis* mc^2^155 cells and electroporated in 0.1-cm cuvettes (Lightlabs) using a Bio-Rad Laboratories GenePulser Xcell electroporator (1.8 kV, 10 µF, 600 Ω) (Bio-Rad). Following transformation, cells were resuspended in 1 mL of 7H9 medium and incubated at 37 °C for 1 h. The culture was spun down at 2,000 × *g* for 5 min and 900 µL of the supernatant was discarded. The pelleted transformed cells were then resuspended in the remaining 100 µL of the media and then plated on 7H11 agar supplemented with 5 µg/mL kanamycin. Plates were incubated at 37 °C for up to 5 d, and colony growth was monitored throughout the incubation period.

### Construction of the pExTra Xavia library

Individual Xavia genes were inserted into the pExTra shuttle plasmid under control of the anhydrotetracycline (aTc) responsive *pTet* promoter, with the transcriptional reporter gene *mCherry* positioned immediately downstream of the cloned insert (Fig. 1a; Heller et al. 2022). Xavia genes were PCR-amplified using gene-specific primers (Integrated DNA Technologies) (Supplementary Table 1), Q5 DNA polymerase (New England Biolabs Q5 HotStart 2× Master Mix), and a high-titer Xavia lysate. Gene-specific primers anneal to the first and last 15 to 25 bp of each gene sequence and introduce a uniform ATG start codon (forward primers) or TGA stop codon (reverse primers). Each primer was also designed to contain regions of identity to the pExTra01 plasmid; forward primers contain a uniform ribosome-binding site (RBS) and 5′ 21 bp of homology to pExTra01 downstream of the *pTet* promoter, and all reverse primers contain a separate 5′ 25 bp of homology to pExTra01 upstream of *mCherry* (Supplementary Table 1). Homologous sequences were used to direct insertion of Xavia gene fragments into the pExTra plasmid downstream of the *pTet* promoter by isothermal assembly with the NEB HiFi 2× Master Mix (Heller et al. 2022). Linearized pExTra01 plasmid was prepared for isothermal assembly reactions via PCR (NEB Q5 HotStart 2× Master Mix) of pExTra01 using divergent primers pExTra_F and pExTra_R. Recombinant plasmids were recovered by transformation of *Escherichia coli* NEB5α F′I^Q^ (New England Biolabs) and selected on LB agar supplemented with 5 µg/mL kanamycin. Sequence integrity of all pExTra–Xavia plasmids were confirmed by Sanger sequencing at Azenta using pExTra_universalR and pExTra_seqF primers, and longer inserts were further validated with internal primers provided in Supplementary Table 1. All plasmid inserts matched the published genome sequence.

**Fig. 1.**
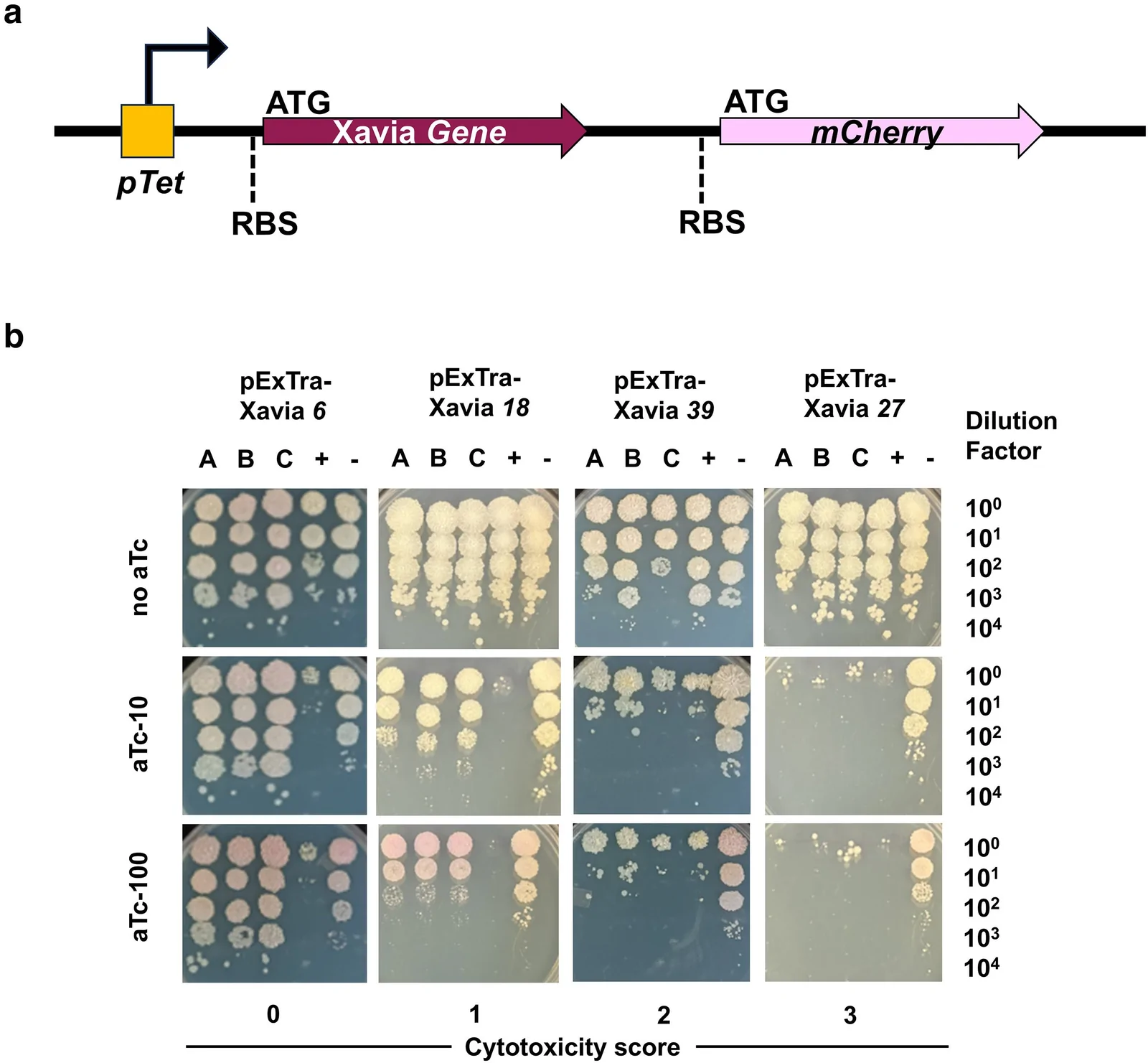
Representative cytotoxicity phenotypes showing Xavia genes with scores 0 to 3. a) pExTra constructs used in this study contain each Xavia gene downstream of the aTc-inducible *pTet* promoter and upstream of *mCherry* in a transcriptionally linked operon. Each open Reading frame contains its own ribosome-binding site (RBS) and translation start and stop codons. b) Representative cytotoxicity assays illustrate the range of observed phenotypes. For each gene, three independent transformants (A, B, C) were grown, serially diluted, and spotted on 7H11 agar containing 0, 10, or 100 ng/mL aTc. Positive control strains carried pExTra02 (Fruitloop 52), and negative controls carried pExTra03 (Fruitloop 52-I70S). Cytotoxicity score 0 is represented by Xavia *6*, score 1 by Xavia *18*, score 2 by Xavia *39*, and score 3 by Xavia *27*. Images shown are representative of at least two independent experiments.

### Cytotoxicity screen and phenotype scoring

Three independent transformed colonies were selected per each gene and serial dilution (10^0^ to 10^−5^) cultures were prepared in 7H9, and 10 µL of each dilution was spotted onto 7H11 plates supplemented with kanamycin and 0, 10, or 100 ng/mL aTc, as described in previous SEA-GENES studies (Heller et al. 2022; Amaya et al. 2023; Pollenz et al. 2024; Tafoya et al. 2025). pExTra-Fruitloop*52* wild type (toxic positive control) and the mutant (I70S; nontoxic negative control) were spot plated in serial dilutions along with the three replicates (Ko and Hatfull 2018; Heller et al. 2022). The growth of *M. smegmatis* was observed over five days. Data quality was assessed using two criteria: control plate growth sufficient to allow internal comparison across the dilution series, and aTc-dependent growth reduction in the positive control strain (pExTra02) within the same experiment, confirming successful induction. Cytotoxic phenotypes were categorized using the scoring framework established in earlier SEA-GENES studies of Amelie, Hammy, Waterfoul, and Girr (Heller et al. 2022; Amaya et al. 2023; Pollenz et al. 2024; Tafoya et al. 2025). Strains showing growth comparable to the negative control were assigned a score of 0. A visible reduction in colony size was scored as 1. A decrease of roughly 1 to 2 orders of magnitude in colony number was scored as 2. A loss of 3 logs or more in recoverable colonies, or the near absence of growth, was scored as 3. For constructs assigned score 0, the presence of pink colonies on aTc plates confirmed induction of the *pTet* promoter and successful *mCherry* expression.

Each gene reported as cytotoxic was assessed in at least two independent experiments. Final cytotoxicity scores were assigned conservatively based on the results observed across all experiments performed on aTc 100 ng/mL. Where independent experiments produced discordant scores, the lowest observed score was assigned as the final cytotoxicity designation. For each cytotoxic gene, two independent representative experiments meeting these criteria are presented in Supplementary Fig. 1; for noncytotoxic genes, one representative experiment is shown. The complete experimental dataset is accessible through the SEA-GENES project open access database GenesDB (https://genesdb.org).

Pink coloration arises from transcription of the downstream *mCherry* reporter and reflects promoter induction by aTc. Thus, pink colonies on aTc plates confirm successful transcriptional activation of the *pTet*–Xavia gene–*mCherry* cassette, even when no cytotoxic phenotype is observed. Occasional weak *mCherry* fluorescence was observed on plates without aTc due to basal promoter leakiness, a phenomenon reported in Waterfoul, Hammy, and Girr screens (Heller et al. 2022; Amaya et al. 2023; Pollenz et al. 2024). This leakiness was not sufficient to affect scoring but confirmed promoter activity.

### Xavia genomic analysis

Gene sequences and annotated functions were taken from the published Xavia GenBank record (accession MH230879). Gene content similarity between Xavia and other phages was quantified using the PhagesDB gene content tool (https://phagesdb.org/genecontent/). Phamily assignments were downloaded from the PhagesDB database on 28 September 2025 and used to compare the distribution of shared and unique gene groups. The Xavia genome map was generated using Phamerator (Cresawn et al. 2011), which displays coding regions, functional annotations, and shared phamilies with related phages.

Functional predictions for Xavia genes were further explored using BLASTp (NCBI) to identify related sequence homologs (hits with E-values < 1 × 10^−5^, >50% coverage, and strong percent identity were considered meaningful). Domain architecture was evaluated using HHpred (Gabler et al. 2020) with the PDB_mmCIF70_25_May database. Predicted protein structures were generated using AlphaFold3 (alphafoldserver.com), and structural similarity searches were performed in Foldseek (van Kempen et al. 2024). Transmembrane domains and membrane topology were predicted using DeepTMHMM (Hallgren et al. 2022). Top hits and associated probability, identity, or confidence scores for these bioinformatic analyses are reported for each cytotoxic gene in Supplementary Table 2.

## Results and discussion

### Inducible expression of Xavia genes identifies strongly cytotoxic and lethal activities in *M. smegmatis*

To investigate the cytotoxic effects of bacteriophage Xavia gene on growth of *M. smegmatis*, a total of 71 genes were individually cloned into the pExTra shuttle plasmid (Heller et al. 2022), which contains a *pTet*-inducible promoter and a linked *mCherry* transcriptional reporter gene (Fig. 1a). The resulting recombinant constructs were selected based on kanamycin resistance and verified by sequencing; all verified plasmids were subsequently used to transform *M. smegmatis*. Despite repeated transformation attempts, transformants of *M. smegmatis* harboring pExTra-Xavia *37* could not be isolated, indicating that basal promoter leakiness in the *pTet* system is likely sufficient to prevent transformant growth as has been reported previously in similar screens (Heller et al. 2022). Consistent with this observation, bioinformatic analysis indicates that gp37 harbors a PIN-like domain (HHPRED probability 99.68%) and shares 38% amino acid identity with an annotated PIN-like toxin from *Prescottella equi* (Supplementary Table 2). gp38 is annotated as a putative antitoxin with HHPRED analysis showing a >99.9% probability hit to the VapBC antitoxin protein from *M. tuberculosis*, and AlphaFold3 predicts a high-confidence protein–protein interaction (AlphaFold3 ipTM = 0.87; pLDDT > 90) between gp37 and gp38. Although not directly tested in this study, we hypothesize that gp37/gp38 may act as a typical Type II toxin-antitoxin system and that co-expression of gp38 may suppress gp37-mediated toxicity.

Similarly, transformants of *M. smegmatis* harboring pExTra-Xavia *48* could not be recovered in multiple independent transformation attempts. In one instance, transformants were obtained and exhibited strong aTc-dependent growth inhibition (score 3; Supplementary Fig. 1), consistent with the hypothesis that basal leaky expression from the pExTra plasmid is sufficient to prevent transformant recovery. We note that stop-codon control constructs, in which an early stop codon is introduced after the ATG start codon to abolish translation while preserving transcription, were not generated for gp37 or gp48 in this study. Such controls have been used in previous SEA-GENES screens to confirm translation-dependent lethality and are recommended as a priority for future mechanistic work on both genes.

For the remaining 69 genes, three independent transformed colonies per gene were suspended in 7H9 broth, serially diluted, and spotted onto 7H11 agar supplemented with 0, 10, or 100 ng/mL aTc. Cytotoxicity scores ranged from 0 to 3 based on colony size and log-fold reductions in viability. Strains expressing the pExTra plasmid carrying the cytotoxic Fruitloop *52* gene (Ko and Hatfull 2018) served as the positive control, while strains expressing the pExTra plasmid encoding the nontoxic Fruitloop *52*-I70S (Heller et al. 2022) were used as the negative control, and both controls behaved consistently across experiments.

Cytotoxicity scores were assigned by comparing growth of strains on 100 ng/mL aTc plates relative to the no-aTc control, following the scoring framework established in previous SEA-GENES screens (Heller et al. 2022; Amaya et al. 2023; Pollenz et al. 2024). Representative genes for each score 0 to 3 are shown in Fig. 1b. Induction with 10 or 100 ng/mL aTc did not alter colony size or numbers for Xavia *6* (score = 0). For Xavia *18*, induction with 10 or 100 ng/mL aTc reduced colony size (score = 1), whereas induction of Xavia *39* reduced colony numbers by approximately 1 to 2 orders of magnitude (score = 2) and induction of Xavia *27* with 10 or 100 ng/mL aTc caused ≥ 3-log reductions in colony numbers (score = 3). For those genes that were scored as cytotoxic, growth inhibition was confirmed in at least one additional independent experiment, with final scores reflecting the most conservative phenotype observed across all experiments, as described in the Materials and Methods and as done in other SEA-GENES screens (Heller et al. 2022; Amaya et al. 2023; Pollenz et al. 2024; Tafoya et al. 2025).

### Inducible expression of Xavia genes identifies cytotoxic and noncytotoxic activities in *M. smegmatis*

In addition to the nontransformable genes *37* and *48,* our cytotoxicity screening identified 18 Xavia genes that inhibited *M. smegmatis* growth in an aTc-dependent manner to different degrees, bringing the total up to 20 cytotoxic genes. The proportion of cytotoxic genes in the Xavia genome was 28%, which is similar to frequencies reported in Hammy (25%) (Amaya et al. 2023), Girr (28%) (Pollenz et al. 2024), Amelie (34%) (Tafoya et al. 2025), and Waterfoul (34%) (Heller et al. 2022).

Ten of the 18 genes caused moderate to severe reduction in viable colony numbers, with two genes (*27* and *51*) impairing colony formation by three orders of magnitude or more (Fig. 2, Table 1). The 18 identified cytotoxic gene products belonged to multiple functional categories. Five were annotated as structure or assembly proteins, including terminase small subunit gp1, portal protein gp3, capsid maturation protease gp4, scaffolding protein gp5, and minor tail protein gp18. Although originally annotated as a minor tail protein, the predicted transmembrane protein Xavia gp29 is encoded within the Xavia lysis cassette, drawing a functional parallel to the cytotoxic lysis-associated TMD protein LysF1b identified in Girr (Pollenz et al. 2024, 2026). Lysin B protein gp27, also within the lysis cassette, was found to be highly cytotoxic (score 3; Fig. 2). Three proteins, DNA methylase gp30, RecT-like protein gp54, and RusA-like resolvase gp55, have putative roles in DNA replication and metabolism. Several other products have predicted roles in the lysis/lysogeny life cycle decision, including antirepressor gp42, excise protein gp43, and immunity repressor gp39.

**Fig. 2.**
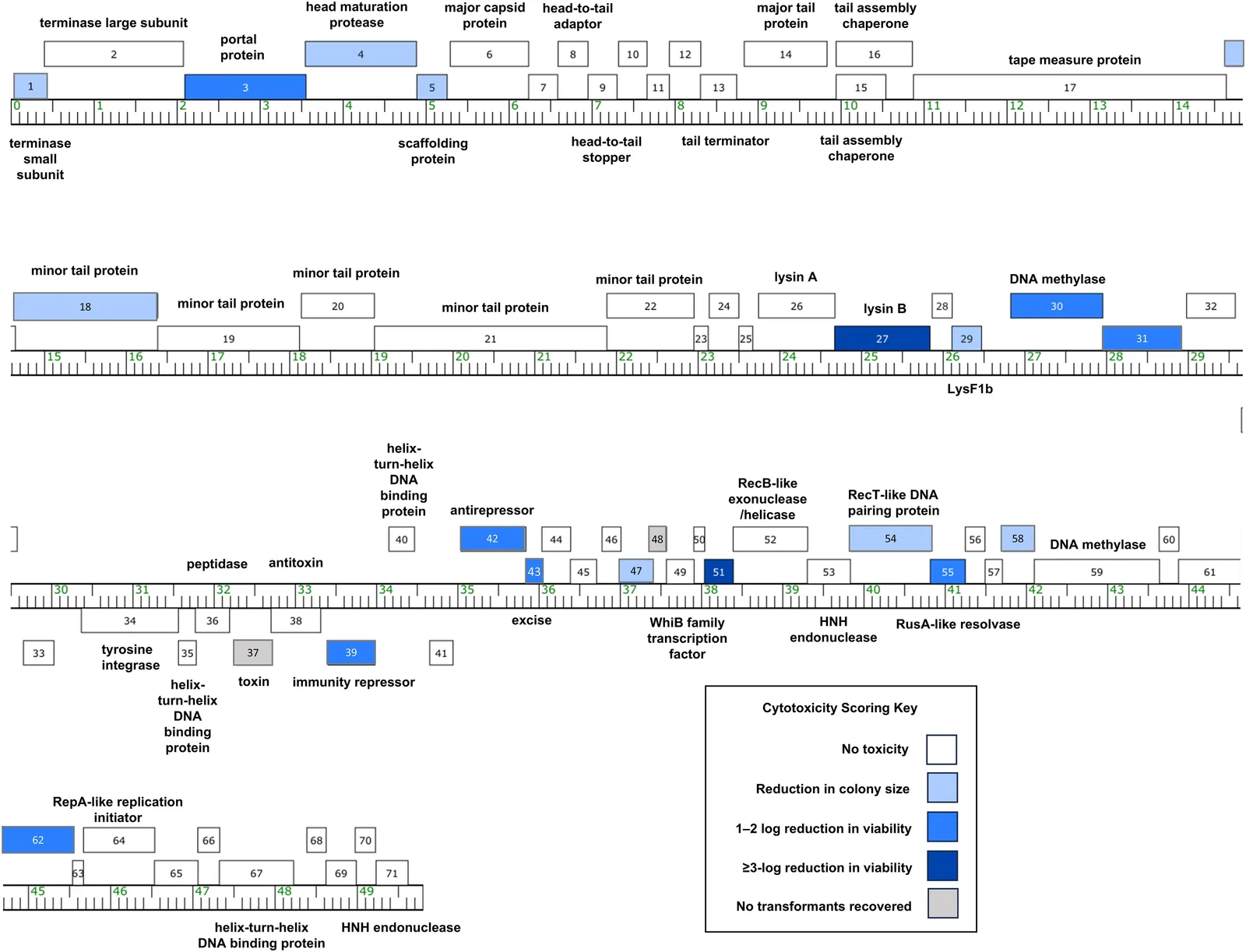
Annotated genome map of mycobacteriophage Xavia with cytotoxicity scores. The Xavia genome is shown with kilobase markers, and each predicted gene is represented by a box, with genes above the line transcribed rightward and those below the line transcribed leftward. Numbers inside each box correspond to gene numbers, and functional assignments follow the Xavia GenBank record and the additional analyses presented in this study. The map was generated using Phamerator. Box shading indicates cytotoxicity scoring, with white boxes designating genes that did not alter *Mycobacterium smegmatis* growth (score 0). Increasing shades of blue indicate greater inhibition: light blue (score 1; reduced colony size), medium blue (score 2; 1 to 2 log reduction in viability), and dark blue (score 3; ≥3-log reduction in viability); gray, no transformants recovered (NT). All cytotoxic genes are individually labeled with gene number and predicted function; cytotoxic NKF genes are color-coded only; noncytotoxic genes are labeled with gene number only. A scoring key is provided in the figure.

**Table 1.** Cytotoxic Xavia genes identified by heterologous expression in *M. smegmatis.*

Xavia gene	Phamily*^a^*	Length (aa)	Predicted function and features	Cytotoxicity*^b^*	Homologs in other phages*^c^*
1	312996	126	Terminase small subunit	1	
4	665	446	Head maturation protease	1	
5	258736	121	Scaffolding protein	1	
18	59	581	Minor tail protein	1	Girr gp15 (Pollenz et al. 2024)
29	261076	120	1 TMD lysis cassette protein LysF1b	1	Girr gp35 (Pollenz et al. 2024, 2026)
47	261119	119	NKF	1	
54	307898	329	RecT-like DNA pairing protein	1	
58	85836	131	NKF	1	
3	130463	486	Portal protein	2	
30	14397	375	DNA methylase	2	
31	14469	321	NKF	2	
39	259157	192	Immunity repressor	2	
42	260356	265	Antirepressor	2	
43	261346	66	Excise	2	
55	253641	143	RusA-like resolvase	2	
62	200918	297	NKF	2	
27	261033	388	Lysin B	3	
51	106454	118	NKF	3	
37	110332	144	Toxin in toxin/antitoxin system	3*^d^*	
48	115328	69	NKF	3*^d^*	

Cytotoxicity was measured by comparing growth of each pExTra–Xavia strain on 7H11 agar containing 0, 10, or 100 ng/mL aTc. Scores reflect reproducible reductions in colony size or viability relative to the same strain plated without inducer. Functional annotations and phamily assignments were obtained from the Xavia GenBank record, Phamerator, and PhagesDB, and were further refined by the HHpred, DeepTMHMM, and AlphaFold3 analyses presented in this study.

^*a*^Phamily numbers were obtained from PhagesDB (accessed 28 September 2025).

^*b*^Cytotoxicity scores reflect reproducible growth inhibition at 10 or 100 ng/mL aTc: 0, no effect; 1, reduced colony size; 2, ∼1 to 2 log reduction; 3, ≥3-log reduction.

^*c*^Homologs shown correspond to genes in previously screened SEA-GENES phages.

^*d*^Transformants could not be reliably recovered for genes *37* and *48*; this untransformable phenotype is distinct from the 0 to 3 cytotoxicity scoring scale applied to all other genes and reflects lethality under basal *pTet* promoter leakiness. NKF indicates a gene product with no known function.

To search for additional functional clues among cytotoxic NKF proteins, we performed HHpred searches against the PDB_mmCIF70 database and examined DeepTMHMM and AlphaFold3 predictions; results for all cytotoxic genes are summarized in Supplementary Table 2. For genes with existing annotations, these structure-based searches supported the assigned roles in structural assembly, lysogeny, and DNA metabolism. Among the 6 cytotoxic NKF proteins, gp47 harbors a DUF732 domain and a predicted signal peptide by DeepTMHMM, suggesting possible extracellular targeting, though its function remains unassigned. gp51 is predicted to contain 2 N-terminal transmembrane domains by DeepTMHMM, suggesting membrane association. gp58 is predicted by AlphaFold3 to adopt a compact mixed alpha-beta fold in its C-terminal region (pTM = 0.7). gp51, gp58, and gp62 returned confident HHpred hits only to other uncharacterized proteins, and gp31 returned no high-confidence predictions by any method. None of these NKF genes have reported cytotoxic effects in previously screened SEA-GENES phages, making them candidates for future mechanistic investigation.

The remaining 51 genes (72%) of the genes tested did not produce cytotoxicity under the overexpression conditions used in this assay, as shown in Fig. 2 and Supplementary Fig. 1. Most of the noncytotoxic genes, 42 out of 51, developed pink colonies after aTc induction, indicating that the gene operon was expressed. Pink coloration reflects promoter activity but does not ensure that the proteins accumulated to levels required for functional effects. The absence of cytotoxicity most directly indicates that expression of a given protein does not impair host growth under the overexpression conditions used in this assay. In some cases, noncytotoxic outcomes could additionally reflect limited protein accumulation, poor solubility, or requirements for combinatorial phage protein activity (Cahill and Young 2019; Mohanraj et al. 2019). Additional validation at the protein level is needed to determine whether these genes encode stable proteins expressed at sufficient levels to affect growth.

### Comparative analysis shows limited phamily conservation but shared cytotoxic functions among mycobacteriophages

To assess whether the cytotoxic genes identified in Xavia share phenotypic similarities with related phages, we compared the corresponding phamilies encoded in Hammy, Waterfoul, Girr, and Amelie (phagesdb.org). Xavia shares 13.14%, 6.77%, 7.42%, and 4.94% gene content similarity with Girr, Amelie, Waterfoul, and Hammy, respectively, resulting in a limited number of shared phamilies. Among the compared phages, only Girr encoded phams related to cytotoxic proteins identified in Xavia. The corresponding phamily member for Xavia gp18, minor tail protein Girr gp15, was not cytotoxic; the two proteins share only 60.33% amino acid identity, suggesting that sequence divergence within this phamily may underlie the difference in cytotoxic phenotype, though the molecular basis of this divergence remains to be determined. On the other hand, the Xavia gp29 phamily homolog Girr gp35, was highly cytotoxic (Pollenz et al. 2024). Both Xavia gp29 and Girr gp35 are encoded in the lysis cassette downstream of predicted holin proteins and harbor a single predicted transmembrane domain, though Xavia gp29 is predicted to also encode a signal peptide immediately upstream of the TM domain. Further characterization of Girr gp35 indicates it plays a critical role in Girr-mediated lysis of the mycobacterial cell (Pollenz et al. 2026), and the cytotoxicity results presented here are consistent with a conserved function in phage Xavia.

Given the low level of gene content similarity between Xavia and previously screened genomes, we also looked more generally for consistent functional themes. The scaffolding proteins Girr gp5 and Amelie gp8 (Pollenz et al. 2024; Tafoya et al. 2025) produced similar cytotoxic effects as did minor tail proteins Girr gp19, Amelie gp20, and Waterfoul gp21 (Heller et al. 2022; Pollenz et al. 2024; Tafoya et al. 2025). More recently, portal protein cytotoxicity was reported for Cluster L1 phage LeBron (Cagang et al. 2026), suggesting this phenotype extends across genetically distinct phage clusters. Conserved roles in DNA metabolism also show up, with DNA methylase Girr gp64 (Pollenz et al. 2024) and RusA-like resolvase Amelie gp58 (Tafoya et al. 2025) exhibiting toxicity. Toxicity of the Xavia immunity repressor gp39 and antirepressor gp42 match observations from Girr showing that its immunity repressor, gp46, and antirepressor, gp48, both of which belong to different gene phamilies than the Xavia products, are highly cytotoxic when overproduced (Pollenz et al. 2024). Immunity repressors from Cluster K phages Hammy, Waterfoul, and Amelie do not cause cytotoxicity, indicating that this phenotype is specific to certain phamilies and not a general consequence of immunity repressor function.

There were some notable differences between Xavia and the other datasets as well. For example, the major capsid proteins from all Cluster K and Cluster F phages tested to date have exhibited toxicity, whereas Xavia gp6 was not observed to inhibit host growth. Furthermore, although multiple lysin A genes have been reported to be toxic when overexpressed individually (Amaya et al. 2023, Pollenz et al. 2024), to the best of our knowledge, this is the first report of lysin B overexpression impairing host physiology. HHpred analysis reveals that Xavia gp27 carries an N-terminal region (residues 5 to 73) with structural similarity to an N-acetylmuramyl-L-alanine amidase fold (3D2Y_A; probability 95.85%), and a C-terminal region with high-confidence similarity to alpha-beta hydrolase/cutinase-like proteins (probability >99%), suggesting an unusual multi-domain architecture relative to canonical lysin B proteins. Notably, the adjacent gp26 (Lysin A) independently shows structural similarity to the same amidase template through its C-terminal region (residues 182 to 246; probability 94.65%). The mechanistic basis for gp27 toxicity remains unclear; however, the domain organization described here represents a structural observation that warrants dedicated functional characterization in future studies.

Altogether, the current screen identified 20 phamilies that impair bacterial growth when overexpressed, and some of these phamilies appear to be novel in the context of cytotoxicity in *M. smegmatis*. This genome-wide overexpression screen of the P3 cluster mycobacteriophage Xavia provides the first functional characterization reported for a cluster P phage. Despite the low overlap with previously tested gene phamilies in Cluster K and Cluster F phages, the proportion of cytotoxic genes in Xavia, approximately 28% is consistent with frequencies, ranging from 26% to 34%, reported in Girr (Pollenz et al. 2024), Hammy (Amaya et al. 2023), Amelie (Tafoya et al. 2025), and Waterfoul (Heller et al. 2022). Cytotoxic Xavia genes were dispersed among regions associated with structural, DNA metabolism, lysogeny, and NKF functions. We note that genes *27*, *29*, *30*, and *31* form a contiguous block of four cytotoxic genes in the genome map, and that genes *42*, *43*, *47*, *48*, and *51* constitute another region of elevated cytotoxic density (5 of 9 consecutive genes, four with scores ≥ 2). These patterns of local enrichment may reflect genomic modules with coordinated roles in host interaction, although establishing functional coupling between these genes is beyond the scope of the current screen. These observations support the idea that diverse phage genomes encode a substantial number of proteins capable of impairing host growth when overexpressed, even when their functions are poorly understood. The consistent patterns across independent experiments and agreement with previously reported SEA-GENES datasets support the robustness of the findings. Structural and domain predictions for NKF genes (Supplementary Table 2) suggest that at least some of these cytotoxic proteins adopt folds associated with known cellular functions, even though their specific activities in mycobacteria are still unclear.

## Data Availability

Plasmids generated in this study are available from the authors upon reasonable request. The authors affirm that all data necessary for confirming the conclusions of the article are present within the article, figures, tables, and deposited supplementary material. Supplementary File 1, Xavia_Supplementary_FINAL_AT.docx, has been deposited through Figshare and is available at https://doi.org/10.6084/m9.figshare.32952071. Supplementary File 1 contains supplementary cytotoxicity assay data, representative plate images, cytotoxicity scoring information, oligonucleotide sequences used for cloning and sequencing validation, and supporting bioinformatic analyses. Plasmid sequence validation data, including Sanger sequencing results, are deposited in the SEA-GENES open-access repository GenesDB (https://genesdb.org), along with additional validation cytotoxicity assays. These data are accessible through GenesDB after free user registration and can be located by navigating from the homepage to the appropriate phage entry. The Xavia genome sequence used in this study is available through GenBank under accession number MH230879. All supplementary figures and tables are in included in Supplementary File 1. Supplemental material available at G3 online.
